# Dissection of Functional Residues in Receptor Activity-Modifying Proteins Through Phylogenetic and Statistical Analyses

**DOI:** 10.4137/ebo.s705

**Published:** 2008-04-28

**Authors:** Alfonso Benítez-Páez, Sonia Cárdenas-Brito

**Affiliations:** Bioinformatic Analysis Group—GABi. Centro de Investigación y Desarrollo en Biotecnología—CIDBIO. Bogotá D.C., Colombia

**Keywords:** receptor activity-modifying proteins, functional divergence, evolutionary history

## Abstract

Type I and type-II functional divergences have been stated to highlight specific residues carrying out differential functions in evolutionary-divergent protein clusters from a single common ancestor. Briefly, type I analysis is based on residue constraints reflecting a gain of function just in one cluster of an entire family of proteins; while the type-II approach is based on residue constraints showing a different chemical nature in every cluster of a protein family. This last evidence is understood as differential functionality among clusters. The Receptor Activity-Modifying Proteins constitute a family characterized by its paralogous distribution in vertebrates. They are known as G-Protein Coupled Receptor modulators. Although several studies have determined their involvement in ligand binding, specificity, and enhancement of signal transduction, the responsible residues supporting those functions are unclear. Using different bioinformatic approaches, we predicted residues involved in different RAMP functional tasks. Many residues localized in an extracellular coil of RAMP proteins were predicted to be under functional divergence suggesting a gain of function in their respective proteins. Interestingly, the transmembrane region also showed important results for residues playing relevant roles where most of them showed a biased distribution on the structure. A relevant role was conferred by the enrichment of type-II residues observed in their sequences. We show a collection of residues explaining possible gain of function and differential functionality in RAMP proteins. These residues are still experimentally unexplored with regards to functionality. Finally, an evolutionary history could be discerned. Mainly, the RAMP2 cluster has evolved in a higher manner than other RAMP clusters. However, a deacceleration in the aminoacid substitution rate of RAMP2 was observed in mammals. Such effect could be caused by the co-evolution of ligands and receptors interacting with RAMP2 through evolution and/or the specialization of this cluster in GPCR modulation.

## Introduction

The Receptor Activity-Modifying Proteins [RAMP] are known to modulate G-protein coupled receptors [GPCR] (Mclatchie, 1998). This modulatory role is based on their ability to couple different GPCRs of the calcitonin family inducing a ligand-specificity change in the receptor. The amylin [AMY] receptor is defined by the calcitonin receptor [CR]/RAMP-1 or CR/RAMP-3 heterodimers, the Calcitonin Gene-Related Peptide [CGRP] receptor consists of the Calcitonin Receptor-Like Receptor [CLR]/RAMP-1 heterodimer, and the adrenomedullin [AM] receptor comprises of CLR/RAMP-2 or CLR/RAMP-3 complexes (Bühlmann et al. 1999; Leuthaüser et al. 2000; Muff et al. 1999; Poyner et al. 2002). In addition to couple with CR and CLR receptors, RAMP proteins are able to bind the VPAC-1, [Vasoactive Intestinal Polypeptide Receptor], PTH-1, and PTH-2 [Parathyroid Hormone Receptor] receptors (Christopoulos et al. 2003). RAMP proteins are annotated in the Pfam Database [Pfam id: PF04901] as integral membrane proteins having intracellular protein transport and GPCR signaling regulation activities [http://www.sanger.ac.uk/Software/Pfam/] (Nag et al. 2006). Most proteins belonging to this family have been found in mammals and fishes. Three RAMP isoforms are commonly found in almost all organisms with the exception of *Takifugu obscurus* where seven RAMP isoforms have been annotated [RAMP1 to RAMP5, RAMP2 isoform splits in RAMP2a and RAMPb, and a spliced RAMP3 isoform was reported] (Nag et al. 2006). At the same time, respective considerations for the RAMP nomenclature were defined according to phylogenetic analyses in all clusters of RAMP proteins.

Some sequence analyses have shown several conserved residues in RAMP proteins (Udawela et al. 2004; Hay et al. 2006). In those studies, such residues have been associated with both agonist ligand and receptor binding regions, which were determined in other studies through mutational analyses of human RAMP proteins (Kuwasako et al. 2001; Kuwasako et al. 2003). Recently, a relevant structure-maintaining role was conferred to four strongly conserved cysteine residues in RAMP sequences (Simms et al. 2006). Moreover, phylogenetic analyses have showed evidence that RAMP emerged prior to Euteleostomi [bony vertebrates], and duplication events produced the currently known RAMP isoforms (Nag et al. 2006; Benitez-Paez, 2006). In spite of sequence divergence of the RAMP clusters, many residues show selective constraints in all RAMP sequences analyzed for gnathostomata taxon. This data reveal the possible critical role of these residues within RAMP proteins. On the other hand, it has not been possible to determine the specific residues responsible for the differential ligand-specificity in RAMPs. Because each RAMP cluster is able to differentially bind both ligand and receptor molecules, some specific residues must act to confer the receptor activity-modifying function.

Based on the latter idea, some algorithms have been implemented to determine residues where functional divergence appears after gene duplication (Gu, 1999). Three different site states are analyzed in these models; i) Type-0 sites or residues under functional divergence type-0, where the amino acid configurations are universally conserved through the entire protein family. Such residues are presumably associated with the common function among different clusters of a protein family; ii) Type-I sites or residues under functional divergence type-I, where amino acid configurations are highly conserved in one cluster of the protein family and variable in the others; and iii) Type-II sites or amino acids under functional divergence type-II. In this state, the amino acid configuration is highly conserved in every cluster of a protein family but with radically different chemical properties. This last type has been denoted as cluster-specific functional divergence, and it is reflected by a radical shift of amino acid properties (Gu, 2006).

The statistical models for type-I and II divergence retrieve a site-specific profile based on posterior probabilities or confidence values of predictions. Relevant results were shown in a previous analysis for Caspase proteins. There, some sites showing 0.74 and higher posterior probability values for type-I functional divergence were correlated with experimental evidence of their roles in the three-dimensional structure for ICE [Caspases 1, 4, 5, 11, 12, and 13] and CED-3 [Caspases 2, 3, 6, 7, 8, 9, 10, and 14] subfamilies (Wang and Gu, 2001).

One aim of this work was to identify most RAMP sequences stored in the main biological databases. The latter was required to extract further information about sequence-based functionality. This searching was specifically based on the analyses of ESTs and Genome annotations. After the sequence searching, an extensive phylogenetic analysis was also carried out to establish new considerations in the process of RAMP evolution. Finally, an analysis of type I and II functional divergence was done with the aim of identifying the principal residues involved in both ligand and receptor binding for each RAMP cluster. Because the importance of some amino acids in gene-product function can be measured by functional constraints in protein sequences, the type I and II divergence strategies have been tested in a well known cluster of protein families giving relevant information about their functional evolution after gene duplication (Gu, 2006; Wang and Gu, 2001; Gu, 2001). Our methodology could predict specific residues possibly involved in both the modifying activity on GPCRs and the coupling function of RAMP proteins with their respective receptors.

## Results and Discussion

### Sequence analysis

Following method described here (see methods) to retrieve the greater number of RAMP sequences, it was possible to collect 71 non-redundant RAMP sequences. Most of these sequences were used to retrieve confident results at final stages of this study (information concerning all sequences is listed in Table S1 of supplementary material). RAMP sequences retrieved from genomic and ESTs records were scanned for a specific RAMP pattern [see methods] based on most type-0 residues observed in RAMP proteins. Notably, most reported RAMPs belonged to fishes and mammals. At the same time, it was possible to find more than 3 RAMP isoforms in the fish species *Gasterosteus aculeatus*. Recently, existence of more than 3 RAMP isoforms has been reported in studies where several isoforms of RAMP proteins were reported for *Takifugu obscurus* and *Takifugu rubripes* (Nag, 2006; Benitez-Paez, 2006). All RAMP sequences retained all main conserved residues described in methods section. This observation supports the notion of selective constraints acting on a proportion of residues with structural or functional importance for RAMP family as a whole [those called type-0 residues].

### Phylogeny

**Figure 1:**
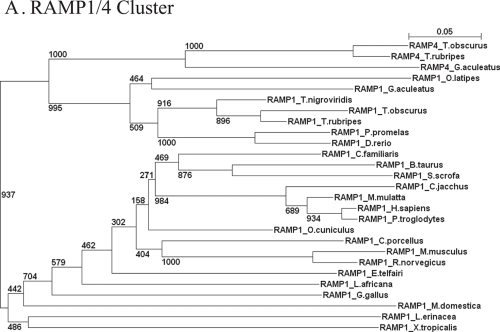

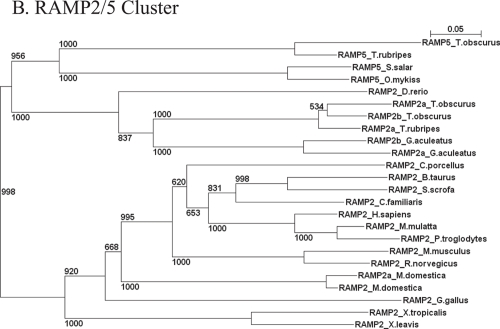

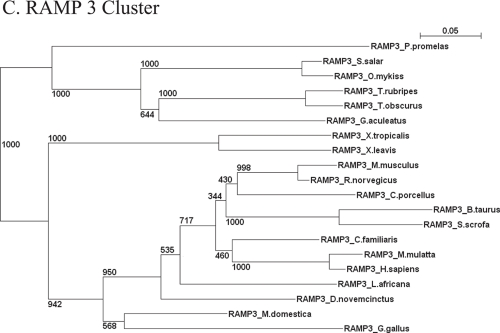
Phylogenetic analyses of separately RAMP clusters Phylogenetic analyses of different RAMP clusters were firstly submitted through the Prottest application (Abascal et al. 2005) to confirm the best model for each cluster. **A**—The bestfit for Cluster RAMP1/4 was the Jones Thorton Taylor model plus Gamma distribution plus amino acids frequencies [JTT+G+F]. **B**—Best model explaining phylogeny of cluster RAMP2/5 was determined by JTT+G one. **C**—Phylogeny in cluster RAMP3 was resolved by JTT+I [Invarianted frequencies]+ G model. Branches reflect phylogenetic distances and bootstraps over 1000 replicates.

After compilation of RAMP sequences in different biological databases, it was possible to perform several phylogenetic analyses to determine the evolutionary relationships of the RAMP family. Figure 1 separately shows the phylogenetic relationships for RAMP clusters. The phylogenetic tree of the RAMP2 cluster shows higher distance values than other clusters, evidence that the RAMP2 cluster has evolved at a higher rate than the RAMP1 and RAMP3 clusters. A basic comparison among branches gives ratios of 1.41 and 1.18 fold for RAMP2/RAMP1 and RAMP2/RAMP3, respectively, in *Homo sapiens* proteins. Similar values are shown in *Mus musculus* and *Rattus norvegicus*. Higher differences were observed in *Gallus gallus, Xenopus* genus, and fishes. For instance, branches ratios of 1.71 fold for fishes and 1.83 fold for chicken in RAMP2/RAMP1 comparison were observed, while RAMP2/RAMP3 comparison showed respectively 1.37 and 1.42 ratios. This slight increasing in branch ratios suggests an accelerated evolutionary process only in fish RAMP2 cluster since differences for RAMP3/RAMP1 comparison were almost identical in all groups before analyzed (1.30 for fish, 1.22 for human, and 1.29 for chicken in RAMP3/RAMP1 pair).

We hypothesize one possible explanation for latter result, it emerges from the whole view of RAMP/Receptors/Ligand evolutionary analysis. Although this issue is not a direct concern of our study, we have noticed that in fishes, such as *Takifugu obscurus* [mefugu], there are many duplications of RAMP2 partners (Nag, 2006). Those events occurred especially in CLR and AM genes producing variable proteins. In mefugu, RAMP2 is able to bind CLR1 and CLR2 giving specificity at least for AM1 and AM2 ligands. In a rapid exploration for the homology among different AM ligands in the close related species to mefugu, *Takifugu rubripes*, a poor similarity was observed. This similarity value does not exceed 18% of overall sequences [data not shown]. Through these observations, we can infer that high variability and evolution rate of RAMP2 may be a response to the high variability in the ligands to which it binds. This idea is consistent with the evolutionary context seen in mammals. For instance, in mammals only one AM protein has been determined to be recognized by just one CLR. Such pattern is correlated with the evolutionary deacceleration observed in RAMP2 proteins through vertebrate evolution.

Additional evidence supporting the high early-evolutionary rate in the RAMP2 cluster is found in the large branch length of the RAMP5 node, which is only present in fishes. Those values are similar to the branch lengths seen for the RAMP2 cluster. Because RAMP5 cluster emerged from an early RAMP2 duplication, this cluster retains the high variability of its ancestor.

**Figure 2:**
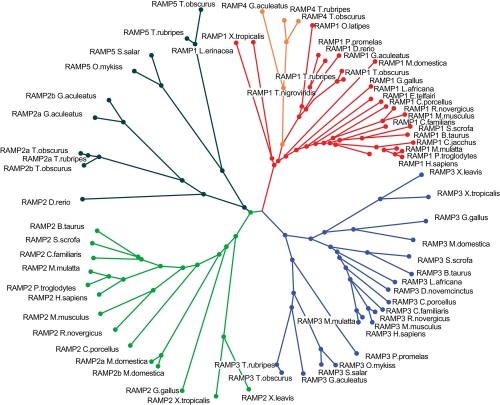
Phylogenetic tree of RAMP proteins Full phylogenetic analysis was done through protest software for all seventy-one RAMP proteins evaluated in this study. The phylogeny model consisted of JTT+G one. Branches reflect only phylogenetic distances based on this model.

Figure 2 shows the full RAMP family phylogeny where the 71 RAMP sequences are depicted in an unrooted tree. Clustering of the three major subfamilies, RAMP1, RAMP2, and RAMP3, is readily apparent. Based on data shown in Figures 1b and 2, the duplication of the RAMP2 subfamily giving origin to RAMP5 apparently occurred before the Tetrapoda lineage. Interestingly, RAMP5 genes had to be lost in ancient fishes prior to terrestrial adaptation. Such loss would be a non-deleterious event given that RAMP5 shows a redundant role with RAMP2a/RAMP2b and RAMP3 proteins for the production of the AM1 receptor in *Takifugu obscurus* (Nag, 2006).

On the other hand, phylogenyetic tree of RAMP1 genes shows a recent origin of the RAMP4 proteins. As a consequence, it is likely to appear RAMP4 genes in organisms other than fishes, but they would not have been identified so far.

### Type-I functional divergence

**Figure 3:**
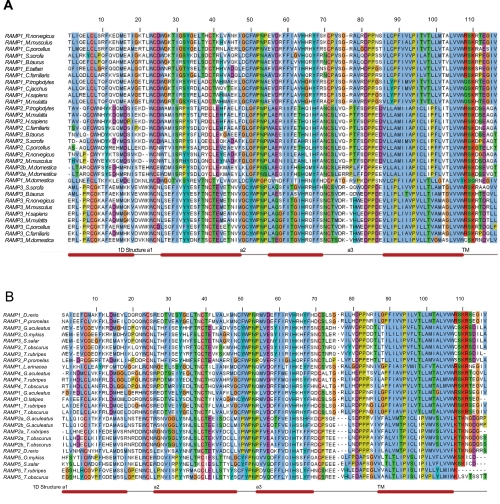
Multiple alignments of RAMP groups RAMP proteins were clustered in two different groups according their vertebrate class origin being mammals or fishes. **A**—Thirty-two mammal RAMP sequences were aligned by T-Coffee software (Notredame et al. 1998). The sequences from *Orictolagus cuniculus* and *Loxodonta africana* were excluded because these partial sequences perturbed analyses. **B**—Twenty-six fish RAMP sequences were aligned as above except the RAMP protein from *Tetraodon nigroviridis*, which was a partial sequence and also disturbed the analyses. Amino acid numeration and secondary structure are based on previous RAMP analyses (Benitez-Paez, 2006).

**Figure 4:**
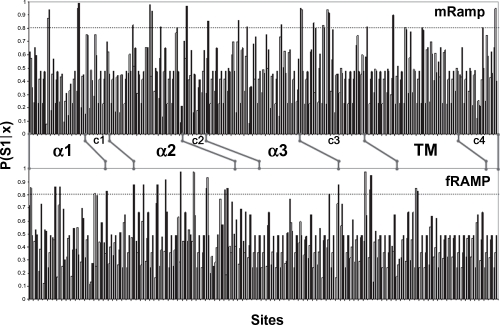
Site-specific profile of Type-I functional divergence of RAMP proteins Multiple alignments of each RAMP group (mammal and fish) and respective phylogenies were submitted to DIVERGE 2.0 software (Wang and Gu, 2001) and Type-I functional divergence analysis was performed. The posterior probabilities were plotted separately for each group. The dashed line shows the threshold value for significant residues under type-I functional divergence. mRAMP = RAMP proteins coming from mammals (specific sequences of some organisms belong to Boreoeutheria subclade of Placentalia Infraclass and Order Didelphimorphia of Marsupialia Infraclass). fRAMP = RAMP proteins proceeded from fishes. White bars show scores for the RAMP1/RAMP2 pairwise comparison; Grey bars show scores for the RAMP1/RAMP3 pairwise comparison; Black bars show scores for the RAMP2/RAMP3 comparison. Secondary structures are defined for each group; non homogeneous distribution was caused by no type-I information for some residues.

With the aim of detecting a possible gain-of-function in each RAMP cluster, a type-I functional divergence analysis was done using the statistical model established by Gu (1999). The analyses were performed separately in fishes and mammals according to the following reasons: i) they were the groups in which more RAMP proteins were recovered, ii) to study separately the evolution of the RAMP proteins in both taxonomic groups provides a comparison to determine either homogeneity or different functional changes through evolution of RAMP clusters, and iii) an initial analysis showed no residues under type-I nor type-II functional divergence when an overall sequence analysis was done. Therefore, fish and some mammal RAMP sequences [belong to Boreoeutheria subclade of Placentalia Infraclass, and Didelphimorphia order of Marsupialia Infraclass, according to Kriegs 2006] were grouped for each analysis. Their respective analyses through multiple sequence alignments are shown in the Figure 3A and 3B. In these alignments the residues corresponding to the extracellular domain, the transmembrane region, and the short intracellular coil are included. Once the type-I functional divergence was done for every group of RAMP proteins, the probabilities in each residue were plotted (Fig. 4). Based on previous data (Wang and Gu, 2001) we limited the prediction for type-I residues with values greater than 0.800. This more conservative threshold was chosen because RAMP proteins structures come from in silico predictions and the functional residues of each RAMP protein are not clearly known. A total of twenty residues showed significant type-I functional divergence in mammalian RAMP proteins while eighteen residues were seen in fishes [Table 1].

The residues under type-I functional divergence are distributed throughout the entire sequences of the RAMP proteins. Furthermore, residues 9, 35, and 75, have shown a similar pattern of functional divergence in mammalian and fish RAMP proteins. Residue 9 was predicted from RAMP3 proteins [Lys mRAMP3/Glu in fRAMP3] and residue 75 was predicted from RAMP1 [Gly in both mRAMP1 and fRAMP1]. Remarkably, the secondary structures showing the highest number of residues under type-I functional divergence are the α2 helix from the fRAMP proteins and the coil linking α3 and transmembrane helices from the mRAMP proteins. Moreover, the main contributions to the pool of type-I residues deduced derive from mRAMP2 [48%] and fRAMP3 [56%] clusters. Additional results show a remarkable divergent functionality in RAMP2 and RAMP3 groups thanks to 71% and 67% of the predicted type-I residues [for mammal and fishes RAMP groups respectively] were retrieved of their pairwise comparison.

The methodology used in this study has supported three out of eight previous hypothesized residues to be involved in ligand and receptor recognition (Benitez-Paez, 2006). In that study, residues 55, 65 and 67, in the α3 helix, were predicted to be involved in the interaction of RAMP proteins with receptors and ligands of Calcitonin family.

Experimental evidence supporting the essential role of that RAMP α3 helix is well known (Kuwasako et al. 2003). However, our data also suggest additional relevant functions in other secondary structures of RAMP molecules. Residues 27 and 31 localized in the α2 helix perhaps function as important sites for ligand binding, while residue 32 could also help in stabilizing the α1/α2 structure given its spatial localization close to the C39 residue [data not shown], which promotes the formation of a disulphide bond with C7 in α1 helix (Simms et al. 2006). These data are also supported by experiments where recombinant human RAMP molecules carrying deletions in these regions decrease the AM affinity of the human CLR receptor (Kuwasako et al. 2001).

**Figure 5:**
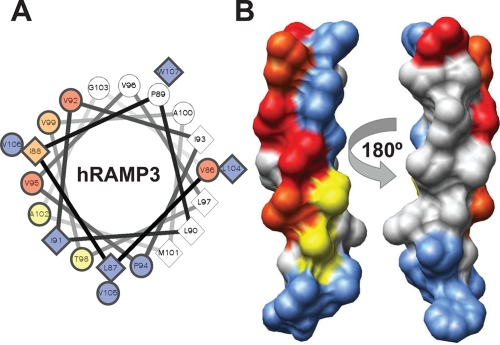
Type-0/Type-I/Type-II residue distributions on transmembrane helix Residues under Type-0, Type-I, and Type-II functional divergences were drawn in a helical wheel plot of the human RAMP3 transmembrane helix. **A**—Helical wheel plot showing Type-0 [blue shaded symbols], Type-I residues [red shaded symbols], and Type-II residues [yellow shaded symbols]; orange shaded symbols represents residues with Type-I and Type-II prediction at same time. **B**—Molecular surface representation of transmembrane helix showing a biased distribution of type-0, type-I, and Type-II residues; color of residues is maintained according to Figure 5A.

Aditionally, type-I residues were predicted in the coil joining the α3 helix and transmembrane structures within the transmembrane region itself, and in the intracellular coil as well. Experimental evidence providing insights for the functionality of that coil of the extracellular RAMP domain has no published at date. Brief analysis of this region was presented by Kuwasako et al., but no relevant results were demonstrated given that most sites under type-I functional divergence came from fixed residues in the RAMP2 cluster (Kuwasako et al. 2001). The Gly75 residue [Gly106 in Kuwasako study] predicted in both mammals and fish was not analyzed in that study, but it seems to be a good candidate given its high degree of conservation in the RAMP1 subfamily. Five residues in the transmembrane region have also shown significant evidence of type-I functional divergence. The localization of these residues on a helical wheel plot and on the structure itself is shown in Figure 5A. In the graph, type-I residues 86, 88, 92, 95, and 99, are distributed on the same side of the transmembrane helix together type-0 residues (i.e. 104–107). This evidence supports the idea a critical role of the transmembrane region in the RAMP/GPCR coupling and stability functions, which seems to be based on several hydrophobic interactions. In fact, the interaction face is composed of nine out of twelve residues showing type-0 and type-I functional divergence, which are disposed in a plane of the helix axis [see Fig. 5]. This biased localization provides good evidence of a direct interaction between the transmembrane region of RAMP proteins and one or more transmembrane segments out of the seven transmembrane helices of GPCRs. Previous experimental results support our theory (Steiner et al. 2002).

Finally, some residues in the intracellular coil were predicted to play a role in mRAMP1 proteins. This role is supported by a recent study (Udawela et al. 2006) where it is shown that attenuated AMY binding occurs when the C-terminus coil is deleted from RAMP proteins. A possible functionality with the α_s_ subunit of the heterotrimeric G protein can also be inferred given that the deleted version of RAMP1 produces a partial recovery of AMY binding when the CR/RAMP receptor is co-expressed with this Gα_s_ subunit. Such an effect is less pronounced in C-terminus deleted versions of RAMP2 and RAMP3. In addition to show a regulatory amino acid pattern [R/K/Q/C]-[S/T]-K-X motif recognized by PKC, the intracellular coil also seems to function in signal transduction by interacting with signalling proteins such as heterotrimeric G protein subunits.

### Type-II functional divergence

**Figure 6:**
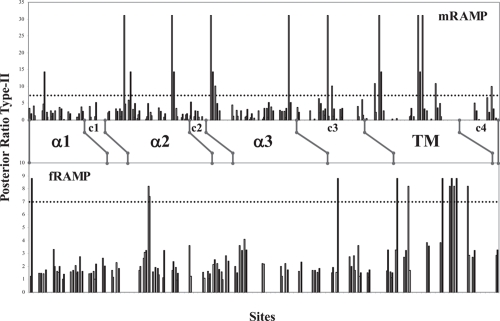
Site-specific profile of Type-II functional divergence of RAMP proteins According to procedure used in type-I analysis, the multiple alignments of each RAMP group were processed by DIVERGE 2.0 (Wang and Gu, 2001) to collect type-II functional divergence data. The posterior ratios retrieved from this analysis were plotted separately for each group. The dashed line shows the threshold value for significant residues under type-II functional divergence. mRAMP = RAMP proteins from mammals. fRAMP = RAMP proteins from fishes. White, grey, and black bars show the scores for the RAMP1/RAMP2, RAMP1/RAMP3, and RAMP2/RAMP3 pairwise comparisons respectively. Secondary structures are defined for each group; non homogeneous distribution was caused by loss of type-II information for some residues.

The type-II functional divergence is based on differential patterns of amino acids that are highly conserved within two different clusters of a protein family but with different chemical properties. This event is also known as a radical shift of amino acid properties in which the change affects the chemistry of the residue and therefore a differential function is deduced from this event. Based on same alignments (Fig. 3), the profiles for type-II posterior probabilities per site are shown in Figure 6. The threshold value using to predict residues under type-II functional divergence was 7. Such value means a higher conservative posterior probability, P(S1|X), equal to 0.875. Lower values were successfully tested in previous analyses of the COX protein family (Gu, 2006). At same time, this value is compatible with no amino acid changes within cluster. Twenty-four type-II residues for RAMP family are listed in the Table 2 (fifteen in mammals and nine in fishes). Residues 29, 89, 98, 99 and 102 were identically predicted in both groups of RAMP proteins. Most of these are present in the transmembrane region. The strongest evidence of type-II functional divergence in both mammals and fishes was in RAMP2 and RAMP1 or RAMP3 clusters, which mean a significant functional divergence in the RAMP1/RAMP2 and RAMP2/RAMP3 pairs. The pairwise RAMP1/RAMP3 comparison showed slight evidence of functional divergence, supported by posterior probabilities lower than 0.875.

In this new analysis, we obtained statistical support for functionality localized in residue 67 of mammalian RAMP proteins. This residue would have a critical role given that it appears under constraint in each RAMP cluster [see Tyr, His, and Phe for RAMP1, RAMP2, and RAMP3, respectively in Fig. 3A]. Residues 77 and 78, belonging to the third coil structure joining α3 and transmembrane helices, also showed strong evidence of type-II functional divergence. These data strengthen and support the functional relevance of this structure that has not been characterized so far. Five out of the approximately fifteen residues belonging to this secondary structure have been predicted under both type-I and type-II functional divergence. In this manner, a promising scenario is providing for further studies based on mutational analyses to confirm their respective roles.

Finally, we focus on the relevant results obtained for residues belonging to the transmembrane domain. Evidence of type-II functional divergence was also collected from this region in both major groups of RAMP proteins analyzed in this study. The role of transmembrane region has been underestimated in an earlier study where, despite expression on the cell surface, cells carrying human RAMP1 with a partial deletion in its transmembrane region displayed weak intracellular response (Fitzsimmons et al. 2003). Unlike Fitzsimmons and et al.’s study, the relevance of the transmembrane region of RAMPs was recognized in another study (Steiner et al. 2002). Steiner et al. showed that partial deletions in the transmembrane domain of RAMP1 induced a marked decrease in signalling mediated by the CGRP receptor. Although a GPCR/RAMP complex stabilizer role was assigned to the RAMP transmembrane region in last study, any basis of that molecular role had not been fully elucidated. Furthermore, four out of seven different type-II residues in transmembrane region [89, 98, 99 and 102] were consistent in both mammalian and fish groups of RAMP proteins. The pattern of these residues in each RAMP cluster shows differences with regard to functionality. Interestingly, type-II residues 88, 98, 99 and 102 of mRAMP co-localize in the structure with most highly conserved residues and those under type-I functional divergence [Fig. 5]. Globally, these data allow us to hypothesize a critical role for the transmembrane region mediating the receptor specificity and directly depending on RAMP molecule coupled to receptor.

## Conclusions

Nowadays, the genomic information available in biological databases allows extracting and understanding functional properties stored in the sequences of a family of gene/proteins. Here, two main groups of RAMP proteins were studied, which 34 RAMP sequences were from mammals and 27 RAMP sequences were from fishes. Both groups of proteins represent 86% of the total number of RAMP sequences studied. Additional RAMP clusters were found for *Gasterosteus aculeatus*, *Salmo salar* and *Oncorhynchus mykiss*. This greater genomic distribution of RAMP proteins was previously seen in other fishes such as *Takifugu rubripes* and *Takifugu obscurus.*

The relationships obtained from phylogenetic analyses provided a basis to determine relevant features of evolutionary rates, duplications, and deletions in th gene family encoding the RAMP proteins. Clearly, the RAMP2 subfamily shows an increased rate of amino acid substitution when compared with the RAMP1 and RAMP3 subfamilies. Such increase was confirmed by the same pattern in the RAMP5 subfamily, which is an early evolutionary product of RAMP2 gene duplication.

In addition, statistical analyses were also performed to shed light on the specific residues playing a critical role in RAMP protein function. A gain of function [Type-I functional divergence] and the functional divergence supported by a radical shift of amino acid property [Type-II functional divergence] per site were evaluated. In the first procedure, twenty residues were predicted to undergo type-I functional divergence in mRAMP while eighteen residues were predicted for fRAMP proteins. These residues were distributed in structured regions of RAMP proteins, but the most significant ones were localized on α2 helix, in the coil linking α3 and transmembrane helices, and in the transmembrane region itself. Some residues predicted under type-I functional divergence and disposed in the α3 helix have been experimentally shown to have functional relevance (Kuwasako et al. 2003). Main residues localized in the coil linking α3 and transmembrane helices have not been confirmed in this manner. We propose that experimental analysis of their function would indeed be interesting. In addition to the highly conserved residues in the transmembrane regions other residues show significant evidence of a gain of function. Most of these were localized on the same side of the helix (Fig. 5). The transmembrane helix seems to have a highly relevant role in both the interaction of the RAMP/GPCR complex.

The process of amino acid fixation in a subfamily of proteins refleacts new adaptative functional roles [positive selection] and explained by type-I functional divergence analysis. This method brings on more information concerning evolution of RAMP proteins. The 50% of type-I residues predicted from mRAMP group were present in RAMP2 cluster, while only 11% was seen in fish RAMP2 cluster. This observed specialization is correlated with the branch length decreasing discerned for these proteins through evolution. The gain-of-function study in some residues of RAMP proteins was complemented by other statistical analyses to predict residues under type-II functional divergence. After identifying many type-I residues that could be carrying out different functions in RAMP proteins in their respective constraints within each RAMP cluster, we focused on type-II residues as main candidates likely to be involved in differential functionality. In last test, fifteen residues in the mRAMP group and nine in fRAMP were determined to be under type-II functional divergence. Nonetheless, five of these residues corresponded to the same sites in both groups of RAMP proteins. Other relevant type-II residues were detected in the coil linking the α3 and the transmembrane helices. These results suggest a relevant role of these structures in RAMP functionality that previously was detected by type-I analysis.

Prediction of type-II residues was significant given that 8 out of 15 residues in mRAMP group had probabilities >0.950 which means a false-positive rate less than 5% [p < 0.05]. From the data retrieved in this type-II analysis we can conclude that the transmembrane region plays a critical role in each different conformation of the AMY, AM or CGRP receptors. This role would be explaining the selectivity of RAMP molecules to couple specific GPCR. This assumption is based on the biased localization of most type-0, type-I and type-II residues in this structure [see Fig. 5].

In this study, many residues declared as type-I or type-II ones had been previously analyzed by experimental strategies (Kuwasako et al. 2001; Kuwasako et al. 2003). In this way, such experimental evidence supports the validity of our results. This data have conferred weight to different methods used here. Similary, it is possible to strengthen the predictions by increasing the threshold used.

A same evolutionary history was seen in both mammalian and fish RAMP proteins being supported by type-I and type-II residues equally positioned residues on the respective sequences. Although in a few cases the residue identity was observed in a similar position [i.e. Gly75], the prevalence of equivalent functional constraints in both groups of RAMP proteins gives relevant data about the structural and functional roles. Therefore, these constraints discerned in mammals and fishes could react with respective counterparts in unexplored calcitonin ligands or GPCRs sequences.

Nowadays, knowing residues under type-I and type-II functional divergence in RAMP proteins, we could correlate them with type-I residues predicted in both the GPCRs and ligands. Another similar strategy would consist to determine type-II residues also in the GPCRs and ligands to elucidate possible interaction sites through *In Silico Two-Hybrid* approaches based on the correlated amino acid substitution patterns. Plainly, the full methodology described here could be implemented for any type of protein family, which has undergone duplication events.

## Methods

Previous methodology was applied in order to find additional RAMP sequences stored as mRNA, genomic DNA, or translated proteins in the main biological databases (Benitez-Paez, 2006). Multiple sequence alignments were performed with T-Coffee software and default parameters (Notredame et al. 2000). The Hidden Markov Model [HMM] profile for the RAMP family was obtained using HMMER v2.3.2 and its default parameters (Eddy, 1998). Searching for mRNA sequences coding RAMP proteins was done through tBlastn, http://www.ncbi.nlm.nih.gov/BLAST (Altschul et al. 1990) using a HMM profile as the query against EST database. EST assembly was assisted by the CLC Gene Workbench v2.0 [http://www.clcbio.com]. Assembled ESTs were translated to their six possible reading frames and a subsequent probing for the following RAMP pattern obtained from HMM profile was done: C-x-M-x-W-C-x-Y-x-C-x-P-N-x-H-x-F-x-C-x-D-P-P-x-P-x-L-V-V-W-R-S-K [where x means one or more variable residues]. Possible partial RAMP sequences were obtained with short amino acid patterns derived from previous sequence. Genome Blast against Ensembl database [http://ensembl.org/index.html] (Birney et al. 2006) was performed using the same HMM profile used in previous steps. RAMP proteins retrieved in this manner were submitted and compared via BLASTP against the non-redundant protein database in order to define their respective isoforms. Then, the isoform of the known RAMP protein that best aligned to each query sequence was assigned. Phylogenetic reconstruction was done with the Neighbor-Joining method (1000 bootstraps) of Phylip package (Felsenstein, 2004) and using the best amino acid substitution model suggested by ProtTest (Abascal et al. 2005). Unrooted-trees images were produced with Treeview [http://taxonomy.zoology.gla.ac.uk/rod/treeview.html] and NJ-Plot (Perriere and Gouy, 1996). Signal peptides were predicted by SignalP server (Emanuelsson, 2007), and then such sequences were ommited in alignment and later analyses. The Type I and II functional divergences were explored in all three RAMP clusters independently, in both fishes and mammals using the statistical methods proposed by Gu (Gu, 1996; Gu, 2006; Gu, 2001) and implemented in DIVERGE 2.0. Plotting of predicted Type-I and Type-II residues was limited to extracellular RAMP domain, the transmembrane region, and the intracellular coli. Helical plotting of the human RAMP3 transmenbrane helix was done through the Helical Wheel Projections server [http://rzlab.ucr.edu/scripts/wheel/wheel.cgi]. Molecular graphics were designed using the UCSF Chimera package from the Resource for Biocomputing, Visualization, and Informatics at the University of California, San Francisco (Pettersen, 2004).

## Supplementary Material

**Table S1:** List of RAMP sequences used in this study.

Organism	RAMP isoforms	Database	Accession number
*Gasterosteus aculeatus*	RAMP1	Ensembl	ENSGACP00000002846
	RAMP2a	Ensembl	ENSGACP00000011240
	RAMP2b	Ensembl	ENSGACP00000011244
	RAMP3	Ensembl	ENSGACP00000002795
	RAMP4	Ensembl	ENSGACP00000019442
*Oncorhynchus mykiss*	RAMP3	Unigene	Omy.22731
	RAMP5	Unigene	Omy.20200
*Salmo salar*	RAMP3	Unigene	Ssa.1442
	RAMP5	Unigene	Ssa.3961 & Ssa.14756
*Pimephales promelas*	RAMP1	Unigene	Ppr.4120
	RAMP3	Unigene	Ppr.6512
*Danio rerio*	RAMP1	TrEMBL	Q6DGA3
	RAMP2	Ensembl	ENSDARP00000055236
*Xenopus leavis*	RAMP2	Unigene	Xl.56245
	RAMP3	Unigene	Xl.54342
*Xenopus tropicalis*	RAMP1	Ensembl	ENSXETP00000023832
	RAMP2	Unigene	Str.8290
	RAMP3	Unigene	Str.22043
*Monodelphis domestica*	RAMP1	Ensembl	ENSMODP00000011016
	RAMP2a	Ensembl	ENSMODP00000018803
	RAMP2b	Ensembl	ENSMODP00000018802
	RAMP3	Ensembl	ENSMODP00000012835
*Dasypus novemcinctus*	RAMP3	Ensembl	ENSDNOP00000000526
*Echinops telfairi*	RAMP1	Ensembl	ENSETEP00000015736
*Loxodonta africana*	RAMP1	Ensembl	ENSLAFP00000005432
	RAMP3	Ensembl	ENSLAFP00000004103
*Oryctolagus cuniculus*	RAMP1	Ensembl	ENSOCUP00000014399
*Rattus norvegicus*	RAMP1	TrEMBL	Q9JMD9
	RAMP2	TrEMBL	Q9JHJ1
	RAMP3	TrEMBL	Q9JMD8
*Mus musculus*	RAMP1	TrEMBL	Q9WTJ5
	RAMP2	TrEMBL	Q9WUP0
	RAMP3	TrEMBL	Q9WUP1
*Homo sapiens*	RAMP1	SwissProt	O60894
	RAMP2	SwissProt	O60895
	RAMP3	SwissProt	O60896
*Pan troglodytes*	RAMP1	GenBank (ref)	XP_516183.1
	RAMP2	Genbank (ref)	XP_511520.1
*Sus scrofa*	RAMP1	TrEMBL	Q867C0
	RAMP2	TrEMBL	Q867B9
	RAMP3	TrEMBL	Q7YS88
*Gallus gallus*	RAMP1	Unigene	Gga.2711
	RAMP2	GenBank (ref)	XP_418143.1
	RAMP3	GenBank (ref)	XP_418881.1
*Cavia porcellus*	RAMP1	SwissProt	Q8R4C6
	RAMP2	SwissProt	Q8R4C5
	RAMP3	SwissProt	Q8R4C4
*Canis familiaris*	RAMP1	Unigene	Cfa.16785
	RAMP2	GenBank (ref)	XP_537636.1
	RAMP3	Unigene	Cfa.29127
*Tetraodon nigroviridis*	RAMP1	GenBank	CAF97199.1
*Macaca mulatta*	RAMP1	Ensembl	ENSMMUP00000041196
	RAMP2	Ensembl	ENSMMUP00000002868
	RAMP3	Ensembl	ENSMMUP00000005103
*Callithrix jacchus*	RAMP1	TrEMBL	Q8SQ15
*Bos taurus*	RAMP1	Unigene	Bt.2331
	RAMP2	Ensembl	ENSBTAP00000026514
	RAMP3	Unigene	Bt.16077
*Oryzias latipes*	RAMP1	Unigene	Ola.4368
*Leucoraja erinacea*	RAMP1	dbEST	CO051467
*Takifugu rubripes*	RAMP1	Ensembl	SINFRUT00000159414
	RAMP2a	Ensembl	SINFRUT00000138238
	RAMP3	Ensembl	NEWSIN-FRUP00000177881
	RAMP4	Ensembl	SINFRUT00000151422
	RAMP5	Ensembl	SINFRUT00000140300
*Takifugu obscurus*	RAMP1	GenBank	BAE45305.1
	RAMP2a	GenBank	BAE45306.1
	RAMP2b	GenBank	BAE45307.1
	RAMP3	GenBank	BAE45308.1
	RAMP4	GenBank	BAE45310.1
	RAMP5	GenBank	BAE45311.1

## Figures and Tables

**Table 1:** Residues under type-I functional divergence in RAMP proteins.

Sites ^[P(S1|X)] > 0.800^	mRAMP			Sites ^[P(S1|X)] > 0.800^	fRAMP		
	P(S1|X) RAMP1/RAMP2	P(S1|X) RAMP1/RAMP3	P(S1|X) RAMP2/RAMP3		P(S1|X) RAMP1/RAMP2	P(S1|X) RAMP1/RAMP3	P(S1|X) RAMP2/RAMP3
9	0.079	0.873	0.938	1	0.717	0.851	0.490
16	0.326	0.953	0.988	8	0.471	0.453	0.860
31	0.824	0.512	0.650	9	0.171	0.722	0.860
35	0.975	0.438	0.926	17	0.808	0.253	0.793
37	0.269	0.571	0.813	19	0.433	0.350	0.828
43	0.701	0.571	0.965	25	0.469	0.468	0.879
48	0.310	0.574	0.853	27	0.262	0.599	0.879
55	0.165	0.695	0.858	30	0.164	0.733	0.879
57	0.326	0.589	0.811	32	0.366	0.917	0.665
65	0.139	0.736	0.823	35	0.628	0.283	0.977
70	0.948	0.936	0.474	38	0.445	0.975	0.638
73	0.839	0.314	0.801	41	0.849	0.933	0.490
75	0.232	0.820	0.801	45	0.840	0.189	0.848
77	0.232	0.941	0.916	67	0.393	0.455	0.805
86	0.582	0.354	0.812	69	0.166	0.730	0.879
92	0.300	0.423	0.900	75	0.445	0.974	0.638
95	0.232	0.805	0.787	79	0.314	0.840	0.947
99	0.232	0.803	0.786	89	0.243	0.849	0.828
113	0.412	0.803	0.497				
116	0.656	0.946	0.400				

Residues predicted to undergo type-I functional divergence are shown separately for mammalian and fish RAMP proteins [mRAMP and fRAMP respectively]. Type-I predicted residues are those scoring >0.800 per comparison. Site numeration is the same as in Figures 3A and 3B.

**Table 2:** Residues under type-II functional divergence in RAMP proteins.

Sites ^[P(S1|X)] > 0.875^	mRAMP			Sites ^[P(S1|X)] > 0.875^	fRAMP		
	P(S1|X) RAMP1/RAMP2	P(S1|X) RAMP1/RAMP3	P(S1|X) RAMP2/RAMP3		P(S1|X) RAMP1/RAMP2	P(S1|X) RAMP1/RAMP3	P(S1|X) RAMP2/RAMP3
8	0.732	0.825	0.935	2	-	0.555	0.898
29	0.969	0.825	-	29	0.891	0.881	-
30	0.857	-	0.935	70	-	0.604	0.898
40	0.969	-	0.935	86	-	0.766	0.898
49	0.969	-	0.935	89	0.891	0.627	-
50	0.910	-	0.833	96	0.794	-	0.898
67	0.969	-	0.840	98	0.891	-	0.898
77	0.969	0.530	-	99	0.891	-	0.898
78	0.910	0.550	0.772	102	0.891	0.741	-
88	0.916	0.694	-				
89	0.969	-	0.935				
98	0.969	-	0.935				
99	0.969	-	0.772				
102	0.916	-	0.780				
115	0.910	-	0.772				

Residues predicted to be under type-II functional divergence are shown separately as in the Table 1. Type-II predicted residues are those scoring >0.875 per comparison. Site numeration is the same as in Figures 3A and 3B. Negative signs indicate that no posterior ratios were retrieved from the comparisons.
